# C–H bond halogenation catalyzed or mediated by copper: an overview

**DOI:** 10.3762/bjoc.11.230

**Published:** 2015-11-09

**Authors:** Wenyan Hao, Yunyun Liu

**Affiliations:** 1 Key Laboratory of the Functional Small Organic Molecules, Ministry of Education, and College of Chemistry and Chemical Engineering, Jiangxi Normal University, Nanchang 330022, P.R. China

**Keywords:** C(sp^2^)–H bond, C(sp^3^)–H bond, copper, halogenation

## Abstract

Carbon–halogen (C–X) bonds are amongst the most fundamental groups in organic synthesis, they are frequently and widely employed in the synthesis of numerous organic products. The generation of a C–X bond, therefore, constitutes an issue of universal interest. Herein, the research advances on the copper-catalyzed and mediated C–X (X = F, Cl, Br, I) bond formation via direct C–H bond transformation is reviewed.

## Introduction

Organohalides are inarguably a class of most useful chemicals owing to their prevalent application in the synthesis of organic products. The versatile reactivity of C–X bonds allowed them to be used as precursors in the construction of natural products, medicinal, functional materials and agricultural chemicals [1–6]. Therefore, the research toward catalytic generation of C–X bonds constitutes a significant issue in organic synthesis. Electrophilic halogenation of electron-enriched arenes [7–9], diazotization/halogenations of anilines [10], and *ortho*-lithiation/halogenations sequence [11] among others are widely used as traditional strategies for creating C–X bonds. However, one or more problems such as poor site-selectivity, reliance on toxic halogen sources, harsh reaction conditions and/or restricted product diversity remain as challenges for these methods.

The transition metal-catalyzed C–H functionalization has recently gained considerable attention in the preparation of numerous organic molecules [12–18]. In this context, significant advances have also occurred in the C–H halogenation catalyzed by different transition metals as Pd [19–23], Rh [24–25], Ru [26], Au [27], Co [28], etc.

As a class of readily available and ubiquitously employed transition metal catalysts, copper catalysts have exhibited tremendous application in C–H bond functionalizations in recent years owing to their distinct advantages such as low cost, high stability and flexible forms of presence [29–31]. In the area of C–H bond halogenation, the copper catalysis also constitutes a major practical option. To show the power of copper catalysis in modern organic synthesis, herein, we would like to highlight the recent progress in the C–H bond halogenation with copper catalysis or mediation.

## Review

### Copper-catalyzed/mediated halogenation of the C(sp^2^)–H bond

#### Halogenation of the arene C(sp^2^)–H bond

**Scheme 1:**
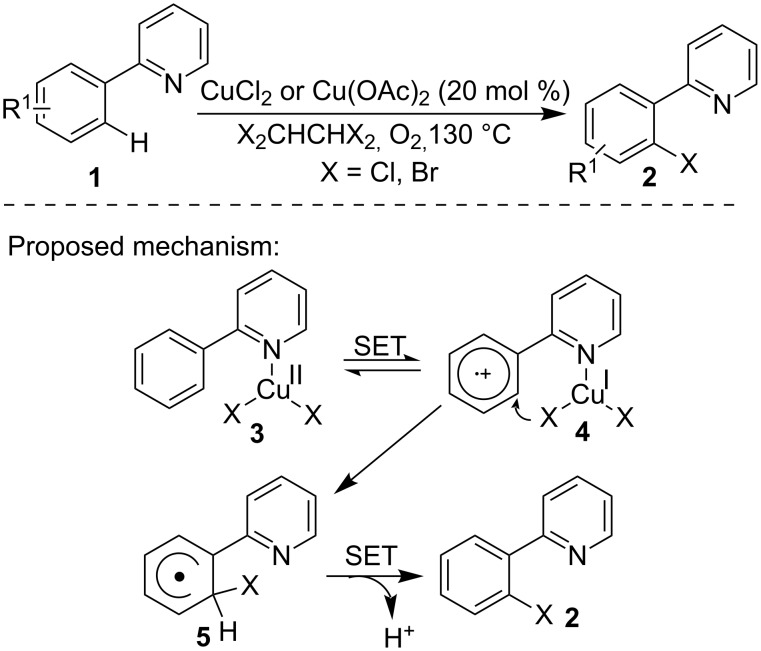
Copper-catalyzed C–H bond halogenation of 2-arylpyridine.

In the synthesis of aryl halides employing the conventional electrophilic halogenation of arenes, the site selectivity was a main challenge and mixed haloarene products were frequently obtained. In 2006, Yu and co-workers [32] first realized the selective *o*-halogenation of pyridine-2-ylbenzenes **1** via C–H activation in a copper/O_2_ system. As shown in Scheme 1, the presence of the pyridine ring was crucial in controlling the site selectivity by forming the copper complex **3** which enabled the selective halogenation of the *ortho* C–H bond of the phenyl ring to give products **2** via transition state **4** and **5**. The 1,1,2,2-tetrahaloethane played the roles of both reaction medium and halogen source. Notably, attempts in the chlorination of the alkene C–H bond under identical atmosphere were not successful. In the reaction process, a single electron transfer (SET) from the aryl ring to the coordinated Cu(II) complex **3** to the Cu(I) species **4** was the rate-limiting step.

**Scheme 2:**
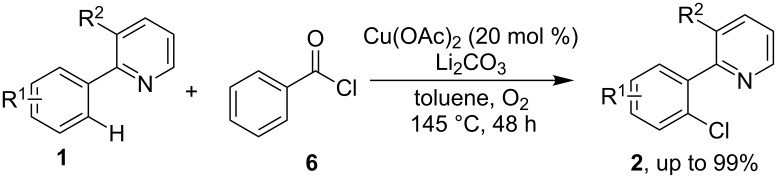
*ortho*-Chlorination of 2-arylpridines with acyl chlorides.

In 2011, Cheng and co-workers discovered an alternative route of a C–H chlorination protocol of 2-arylpyridines by employing acyl chlorides **6** as chlorinating reagents [33]. A range of mono-chlorinated 2-arylpridines **2** were obtained in the presence of Cu(OAc)_2_ and Li_2_CO_3_ under O_2_ atmosphere (Scheme 2).

**Scheme 3:**
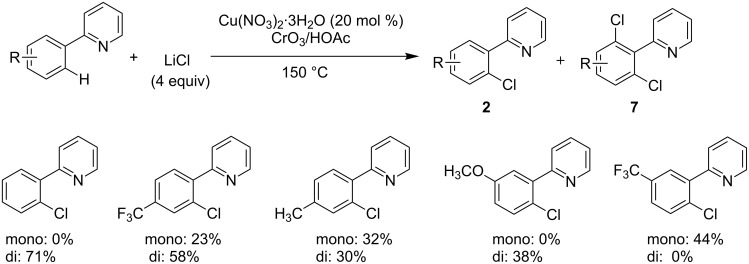
Copper-catalyzed chlorination of 2-arylpyridines using LiCl.

In the same year, Shen and co-workers reported the Cu-catalyzed sp^2^ C–H chlorination of 2-arylpyridines by using the salt LiCl as a new chlorine source in the presence of CrO_3_ and Ac_2_O [34]. Due to the oxidizing potency of the CrO_3_, the application scope of the method was not broad since low selectivity between monochlorinated products **2** and dichlorinated products **7** was suffered (Scheme 3).

**Scheme 4:**
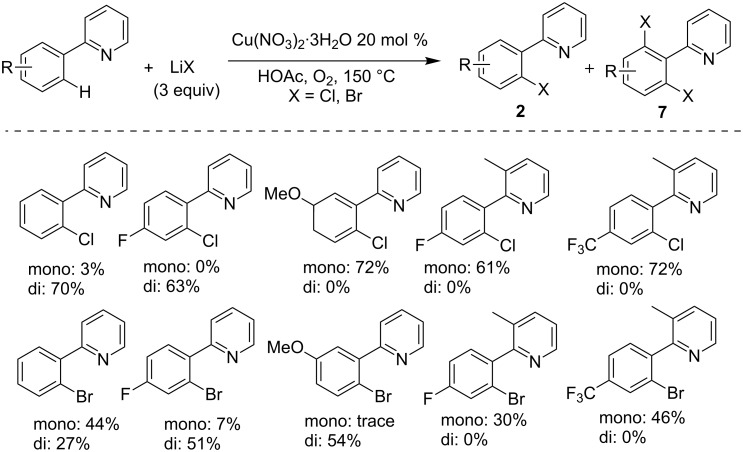
Copper-catalyzed C–H halogenation of 2-arylpyridines using LiX.

Two years later, the same group developed a modified approach for this kind of C–H chlorination by employing lithium halide (LiCl or LiBr) as the source of halogen to react with 2-arylpyridine, which allowed the synthesis of various 2-(*o*-haloaryl)pyridines with improved selectivity towards mono-halogenation in the presence of a copper catalyst [35]. As outlined in Scheme 4, the presence of molecular oxygen as the alternative oxidant enabled most entries providing monohalogenated products with a few examples giving mixed mono- and dihalogenated products.

**Scheme 5:**
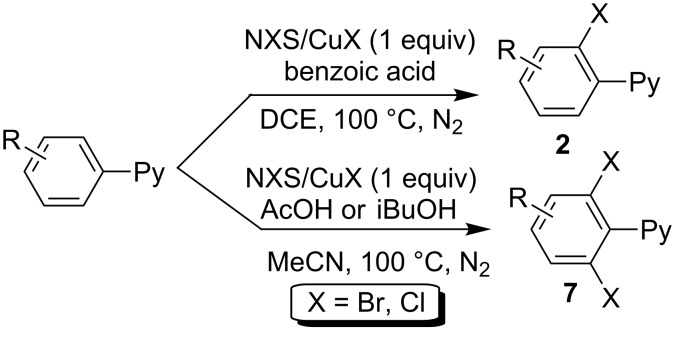
Copper-mediated selective C–H halogenations of 2-arylpyridine.

In all these known arene C–H halogenation protocols with pyridine as directing group (DG), the chemo-selectivity remained as a challenge since either mixed mono- and dihalogenated products or only one of the two potential products could be acquired. In this regard, a synthetic approach allowing the tunable synthesis of mono- and dihalogenated products was highly desirable. Recently, Han and co-workers [36] achieved successfully this kind of tunable reaction via a CuX-mediated aryl C–H halogenation with the assistance of NXS (*N*-halosuccinimide, X = Cl or Br). The application of different acids which participated in the in situ formation of acyl hypohalites enabled the selective generation of products **2** and **7** (Scheme 5). Notably, the C–H iodinated product of type **2** was also observed as key intermediate in the copper-catalyzed pyridinyl-functionalized arene dimerization [37].

**Scheme 6:**
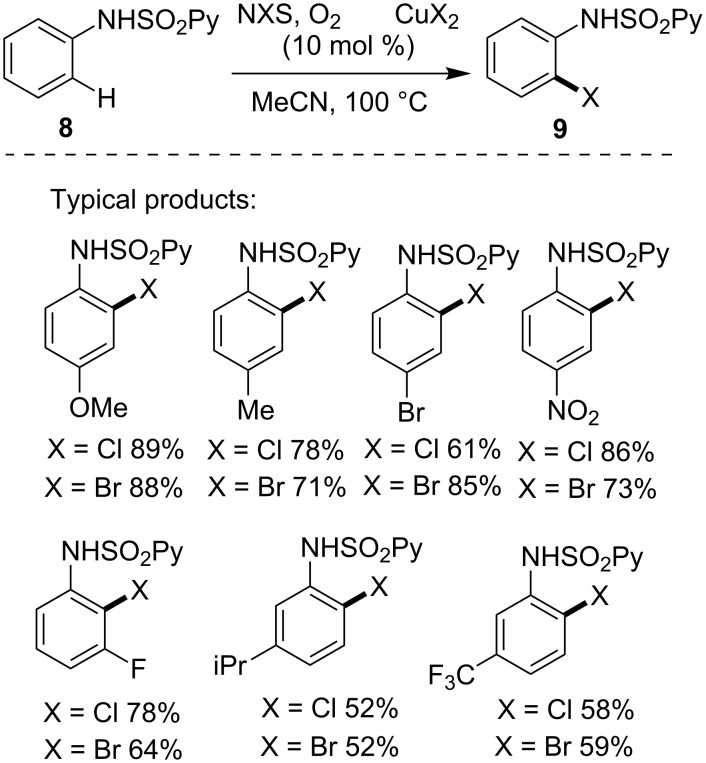
Copper-catalyzed C–H *o*-halogenation using removable DG.

Besides the issue of selectivity, another major challenge in the DG-assisted C–H activation lied in the removal of the DG, which undermined the efficiency of the synthetic procedure. To alternate the hardly removable DG of the pyridine ring, Carretero and co-workers [38] devised a practical copper-catalyzed halogenation of anilines **8** containing an easily removable *N*-(2-pyridyl)sulfonyl auxiliary. In the presence of copper(II) halide catalyst and NXS (X = Cl or Br), a class of *o*-chloro/bromoanilines **9** were efficiently provided under aerobic atmosphere (Scheme 6). The *N*-(2-pyridyl)sulfonyl could be easily removed by treatment with elemental Mg in MeOH.

**Scheme 7:**
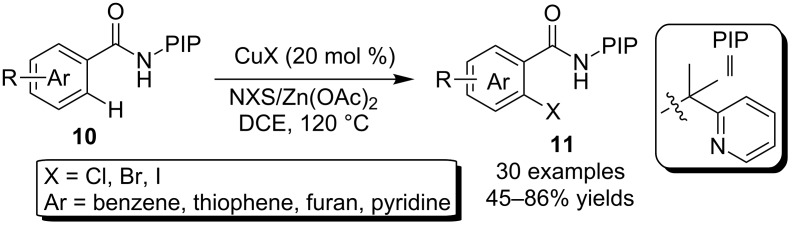
Copper-catalyzed C–H halogenations using PIP as DG.

More recently, Shi and co-workers [39] reported the *ortho*-C–H halogenation of aryl-2-carboxamides **10** using PIP (2-(pyridine-2-yl)isopropylamine) as DG. As shown in Scheme 7, the copper catalyst combined with NXS (X = Cl, Br, I) and a proper additive promoted smoothly the synthesis of various *o*-haloaryl-2-carboxamides **11**. This synthetic protocol tolerated not only carbon aryls, but also various heteroaryls such as thiophene, furan and pyridine in the amide component.

**Scheme 8:**
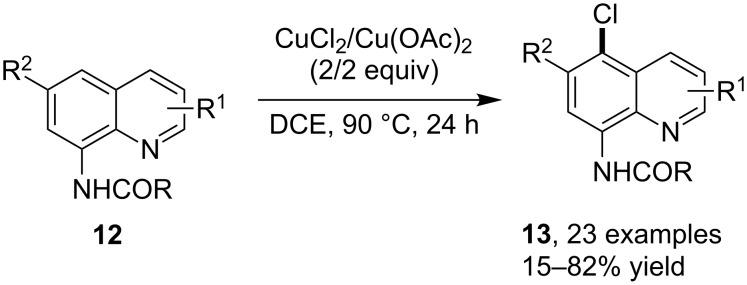
Copper-catalyzed quinoline C–H chlorination.

Interestingly, in an earlier example studying the copper-catalyzed arene C–H methoxylation using the generally applicable quinolin-8-yl [40] as DG, Stahl et al. [41] discovered that using a CuCl/LiCl/O_2_/AcOH catalytic system resulted in the formation of C-5 chlorinated quinoline, demonstrating the pivotal role of the DG in inducing the reaction pathway. These kinds of reactions were later systematically investigated by Xie and co-workers [42]. By using Cu(OAc)_2_ as the catalyst and CuCl_2_ as the chlorine source in DCE, a broad array of 5-chloro-8-acylaminoquinolines **13** were obtained via the selective 5-chlorination of 8-amidoquinolines **12** (Scheme 8). Control experiments in the presence of TEMPO suggested that the reaction might proceed via a C-centered free radical provided by SET between the quinoline substrate and the cupric salt.

**Scheme 9:**
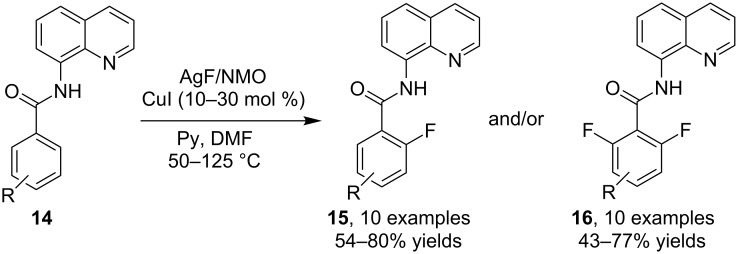
Copper-catalyzed arene C–H fluorination of benzamides.

In recent years, the formation of C–F chemical bonds received global research interest owing to the particular functions of many fluorinated chemicals. Accordingly, C–H fluorination reactions also become an issue of broad concern as such a transformation offers a straightforward route for rapid synthesis of diversity-enriched fluorinated products. On the basis of the Pd-catalyzed oxidative C–H fluorination of functionalized 8-methylquinolinyl substrates reported by Sanford et al. [43], Daugulis and co-worker [44] established in 2013 a copper-catalyzed arene C–H *o*-fluorination of *N*-(quinolin-8-yl)benzamides **14**. The mono- and/or difluorination took place in the presence of CuI, *N*-methylmorpholine *N*-oxide (NMO) and pyridine by using DMF as medium and AgF as fluorine source, providing products **15** and **16**, respectively. As happened in most cases involving the activation of two identical C–H bonds, the unsatisfactory chemo-selectivity in forming mixed products in many entries remained as a problem to address (Scheme 9).

**Scheme 10:**
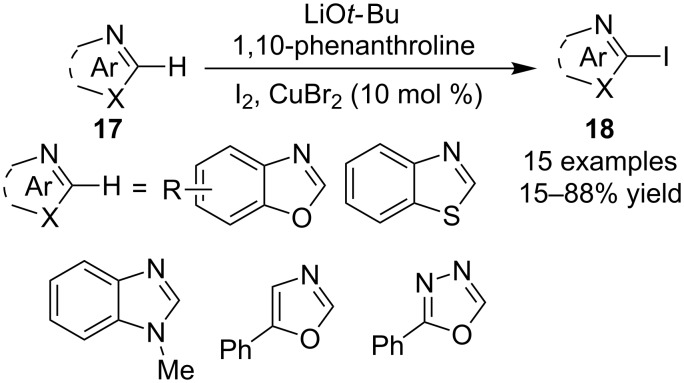
Copper-catalyzed arene C–H iodination of 1,3-azoles.

The catalytic iodination of electron deficient 1,3-azoles was recently realized by Zhao et al. Under the catalytic conditions consisting of LiO*t*-Bu, 1,10-phenanthroline and CuBr_2_, a class of different conventional azoles **17**, including benzoxazoles, benzothiazole, *N*-methylbenzimidazole, 5-phenyloxazole and 2-phenyl-1,3,4-oxadiazole were smoothly iodinated to provide iodoheteroarenes **18** (Scheme 10) [45].

**Scheme 11:**
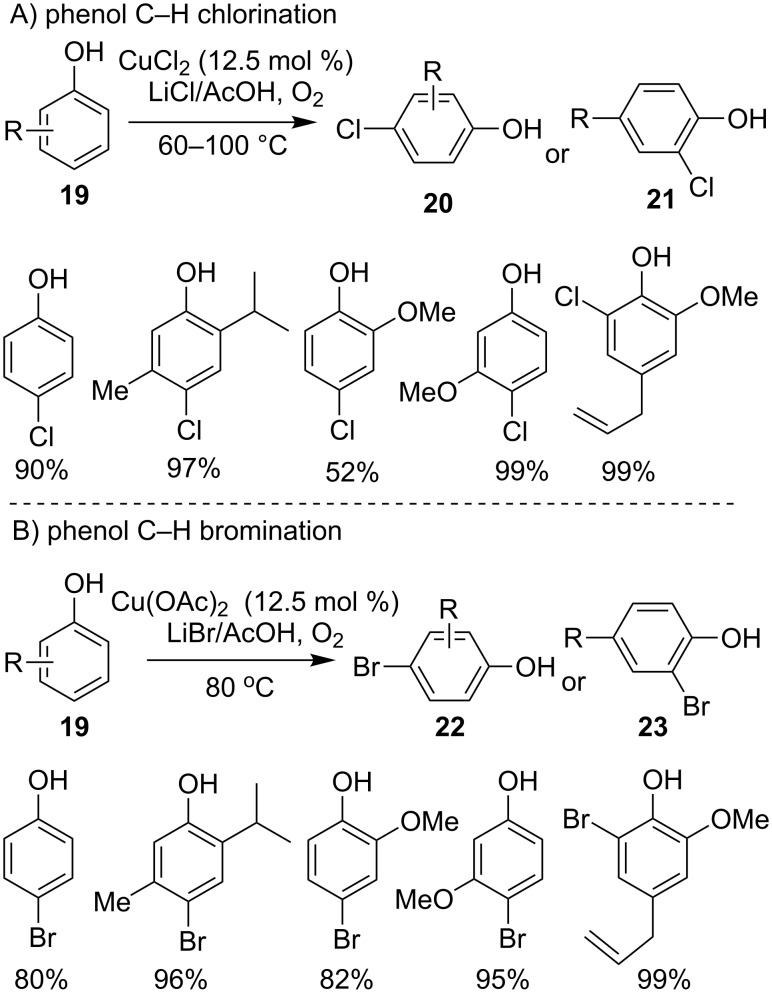
Copper-catalyzed C–H halogenations of phenols.

As typical electron-enriched arenes, phenols and analogous arenes tend to undergo a single-electron transfer process [46], the property of these arenes also resulted in sound attention to their C–H halogenations. In 2006, Gusevskaya and Menini [47–48] reported a highly selective method for C–H chlorination and bromination of various phenols under aerobic, copper-catalyzed conditions. As displayed in Scheme 11, the reaction of phenols **19** with 2 equiv of LiCl in the prensence of O_2_ and 12.5 mol % CuCl_2_ in acetic acid at 80 °C resulted in 93% conversion and 90% selectivity towards 4-chlorophenol **20**, and *o*-chlorinated product **21** was obtained when the *para*-site of the phenol was occupied; analogously, employing LiBr as halogen source led to the formation of equivalent brominated products **22** and **23** under modified catalytic conditions (Scheme 11).

**Scheme 12:**
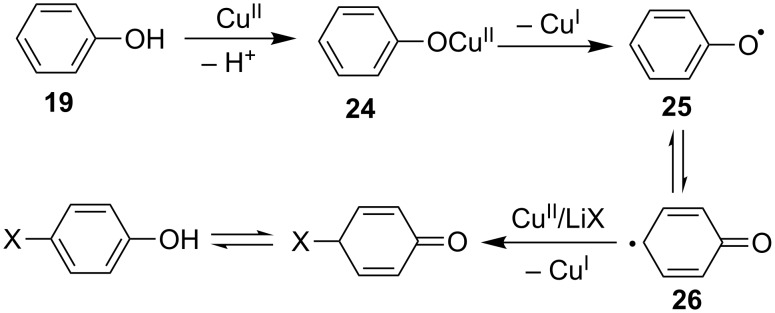
Proposed mechanism for the C–H halogenation of phenols.

However, it was found that the oxidative bromination of phenols exhibited generally lower *para* regioselectivity than the corresponding chlorination [49]. Mechanistic studies on these reactions indicated that the halogenation reactions proceeded via a free radical process (Scheme 12). In the presence of a Cu(II) catalyst, the one-electron oxidation to the phenol led to the occurrence of phenoxy radical **25** via the formation of phenoxyl copper(II) salt **24**. The isomeric free radical species **26** then rapidly captured the halogen atom from LiX to give the target product via oxidation by Cu(II) and release of Cu(I). The presence of molecular oxygen regenerated the Cu(I) species to the Cu(II) catalyst via re-oxidation.

In their subsequent study, Gusevskaya et al. found that anilines were also able to be halogenated via a similar operation. However, the products were acquired as dibromoanilines via double C–H bromination process. On the other hand, the chlorination was found less effective due to the presence of the side transformation and formation of *N*-acetylated byproducts [50].

**Scheme 13:**
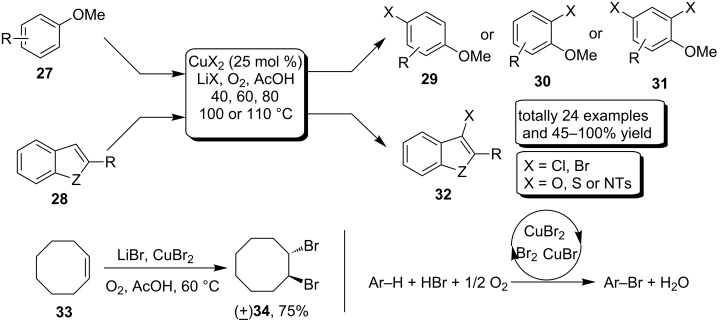
Copper-catalyzed halogenation of electron enriched arenes.

In 2009, Stahl et al. [51] reported a Cu-catalyzed aerobic C–H halogenation protocol of methoxybenzenes **27** and heteroaryls **28**. The method also employed LiX as a halogen sources which led to the production of *para*-halogenated aryls **29** and 3-haloindoles **32**, respectively. Notably, the *para*-substiuted methoxybenzenes provided *ortho*-halogenated products **30**, and employment of excessive LiX provided dihalogenated products **31** (Scheme 13). A preliminary mechanistic analysis suggested that the bromination and chlorination reactions proceeded via different pathways. According the color change in the reaction vessel, the formation of molecular bromine was hypothesized via aerobic oxidation, which suggested the copper-mediated electrophilic bromination as the reaction pathway [52–53]. In addition, the bromination experiment of cyclooctene **33** which yielded *trans*-1,2-dibromocyclooctane **34** also supported the fact that molecular bromine was generated under the catalytic conditions. On the other hand, the chlorination of **33** was not observed under the corresponding chlorination conditions, probably because the decomposition of CuCl_2_ into CuCl and Cl_2_ is much less favorable. The mechanism of the chlorination was not yet clear, but presumably the reaction was initiated by electron transfer from the arenes to the copper-catalyst.

**Scheme 14:**
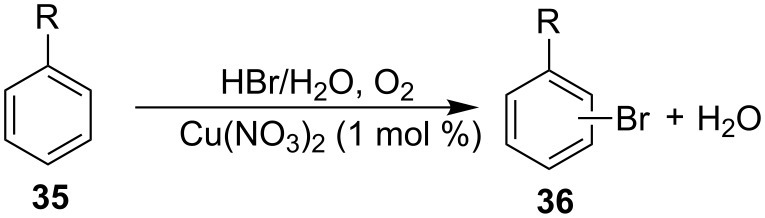
Copper-catalyzed C–H bromination of arenes.

Later on, Li and co-workers [54] developed an effective method of aerobic oxidative bromination of electron-rich arenes **35** by making use of 1 mol % Cu(NO_3_)_2_ as catalyst and 1.1 equiv HBr as additive. Brominated arenes **36** could be acquired with excellent conversions and *para*-bromination selectivity by heating at 100 °C and air atmosphere in water. Simple arenes, including toluene, anisole and cresole were all well tolerated in this transforamtion (Scheme 14).

**Scheme 15:**
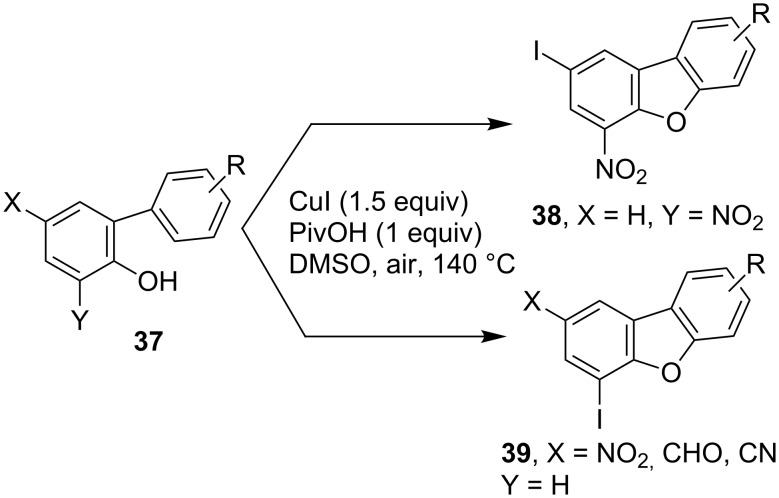
CuI-mediated synthesis of iododibenzo[*b*,*d*]furans via C–H functionalization.

Besides the direct halogenation, a cascade reaction involving a C–H halogenation represents another important approach in preparing C–X containing compounds. Recently, Zhu and co-workers reported a new approach for the synthesis of 2 or 4-iododibenzofurans based on CuI-mediated cascade reactions wherein the C–H iodination and cycloetherification acted as key transformations [55]. As outlined in Scheme 15, in the presence of 1.5 equiv of CuI and PivOH, the EWG-functionalized *o*-arylphenols **37** underwent simultaneous C–H iodination and intramolecular C–H *o*-arylation by heating in DMSO at 140 °C, which led to the production of iodinated dibenzo[*b*,*d*]furans in either the form of **38** or **39** depending on the position of the EWG. The CuI was both the catalyst and the source of iodine in the reactions. It was believed that the iodination took place via in situ generated molecular I_2_ via the oxidation by molecular oxygen since the author successfully observed the presence of I_2_ during chromatography process.

**Scheme 16:**
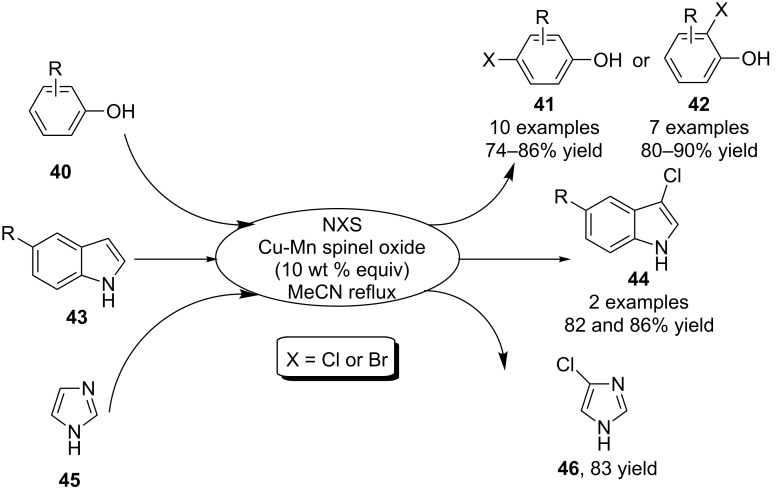
Cu-Mn spinel oxide-catalyzed phenol and heteroarene halogenation.

In the same year, Vishwakarma et al. [56] developed a catalytic approach for the halogenation of phenols and heteroarenes by using reusable Cu-Mn spinel oxide as catalyst. By employing NXS as halogen source, the Cu-Mu spinel oxide was able to catalyze the halogenation of phenols **40** to give either *para*-halogenated or *ortho*-halogenated phenols **41**/**42** in good to excellent yield and regioselectivity. In addition, the C-3 chlorination of indoles **43** and C-4 chlorination of imidazole (**45**) were also achieved under the standard reaction conditions to provide products **44** and **46**, respectively (Scheme 16).

**Scheme 17:**
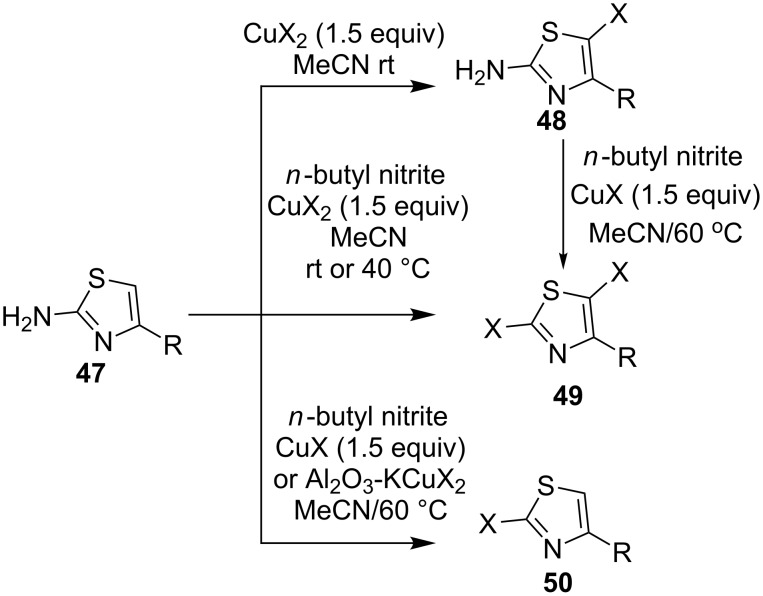
Copper-catalyzed halogenations of 2-amino-1,3thiazoles.

Because of the attractive biological functions of halogenated heteroarenes [57], the synthesis of haloheteroarenes via the corresponding arene C–H halogenations also gained extensive attention. In 2009, Pike and co-workers [58] reported the synthesis of halogenated 1,3-thiazoles using copper(II) halide as a catalyst. As shown in Scheme 17, the catalysis of copper(II) halides allowed selective halogenation of 2-amino-1,3-thiazoles **47** to give 5-halo-2-amino-1,3-thiazoles **48**, 2,5-dihalo-2-amino-1,3-thiazoles **49** or 2-halo-1,3-thiazoles **50** according to the difference on reaction conditions such as temperature, catalyst species etc.

**Scheme 18:**
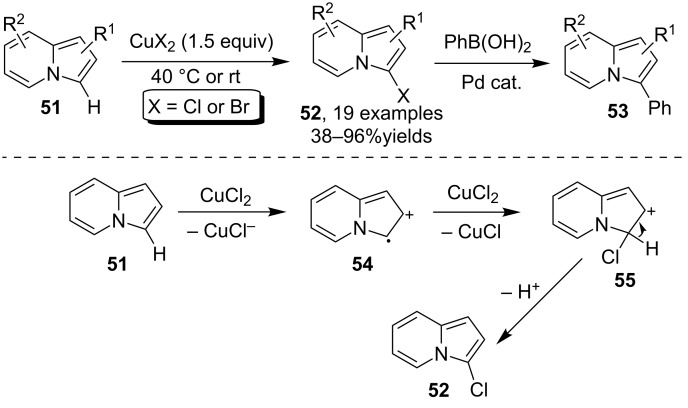
Copper-mediated chlorination and bromination of indolizines.

As a class of structurally interesting fused heterocycles, the indolizines received extensive research interest from the chemical and biological community [59–60]. Owing to the internal reactivity, the arene C–H bond in this arene moiety provided a facile route for elaboration. In 2009, You and Xia [61] disclosed that the Cu(II) halide was able to mediate the chlorination and bromination of indolizines **51** to afford 3-haloindolizines **52** with excellent regioselectivity. The halogenated indolizines **52** were found as highly useful platform compounds for the synthesis of 2-arylated indolizines **53** via Pd-catalyzed Suzuki–Miyaura reaction. The C–H bond activation process was believed to be initiated by the oxidation effect of the Cu(II) catalyst to give intermediate **54** which was further oxidized to provide cation intermediate **55**. The deprotection of **55** gave finally the halogenated products **52** (Scheme 18).

**Scheme 19:**
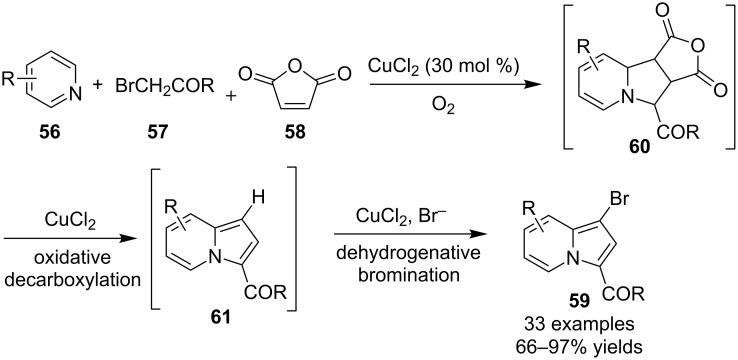
Copper-catalyzed three-component synthesis of bromoindolizines.

Rather recently, Liu and co-workers [62] reported an alternative approach to the synthesis of 3-brominated indolizines via copper-catalyzed three-component cascade reactions of pyridines **56**, α-bromoketones **57** and maleic anhydride (**58**). The construction of the products involved in the three-component annulation intermediate **60** which led to the formation of indolizine **61** via oxidative decarboxylation. And the bromination of **61** took place in situ to give products **59** via an unprecedented dehydrogenative bromination (Scheme 19).

**Scheme 20:**
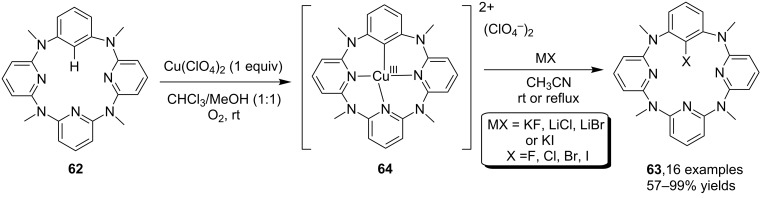
Copper-mediated C–H halogenation of azacalix[1]arene[3]pyridines.

By making use of the copper-mediated arene C–H bond halogenation strategy, Wang and co-workers [63] developed an efficient method for the halogenation of azacalix[1]arene[3]pyridines **62** for the synthesis of halogenated products **63**. The synthesis of the products was mediated by the formation of Cu(III) complex **64**, as observed in the previous study [64], via the assembly of **62** and Cu(ClO_4_)_2_ which enabled the halogenations by using simple alkali salts such as KF, LiCl, LiBr, KI, etc. as the source of halogen (Scheme 20).

Besides in providing various halogenated arene products, the copper-catalyzed arene C–H halogenation had also displayed important application in related C–H transformation by providing key haloarene intermediates. For examples, in the CuI-catalyzed cross arene dimerization reactions reported by Daugulis et al., the in situ formation of iodoarene intermediate was discovered as the indispensable step during the generation of the biaryl products [65].

#### Halogenation of the alkene C(sp^2^)–H bond

**Scheme 21:**
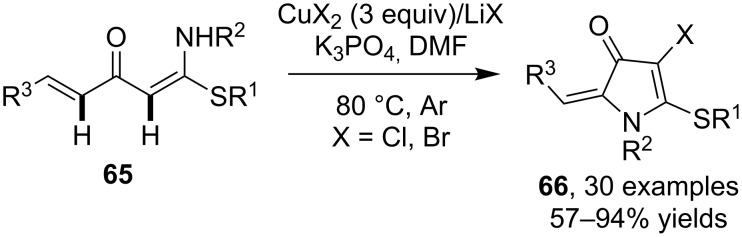
Copper-mediated cascade synthesis of halogenated pyrrolones.

Beside the arene C–H bond, the alkene C–H is another typical C(sp^2^)–H bond. However, unlike the arene C–H bond, the alkene C–H bond tends to undertake difunctionalization via the cleavage of the π-bond in the presence of halogen source. Therefore, the halogenation of alkenes via C–H cleavage is much less known in literature. In 2014, Yu and co-workers [66] reported the cascade synthesis of functionalized pyrrolones **66** via the dual C–H functionalization of α-alkenoylketene *N,S*-acetals **65**. The construction of the products involved the oxidative alkene C–H amination and alkene C–H chlorination in the presence of Cu(II) halide by using LiX as the halogen source (Scheme 21).

**Scheme 22:**
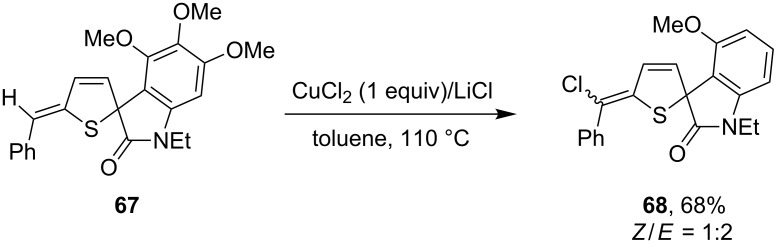
Copper-mediated alkene C–H chlorination in spirothienooxindole.

An earlier example of Cu-promoted alkene C–H halogenation was reported by Jiang et al. [67]. In their investigation to the transformation of α-thienylcarbinols, one of the products spirothienooxindole **67** was found to be capable of undertaking a formal alkene chlorination to provide chlorine-functionalized product **68** as mixed *Z*/*E* isomers (Scheme 22).

#### Halogenation of the C(sp^3^)–H bond

**Scheme 23:**
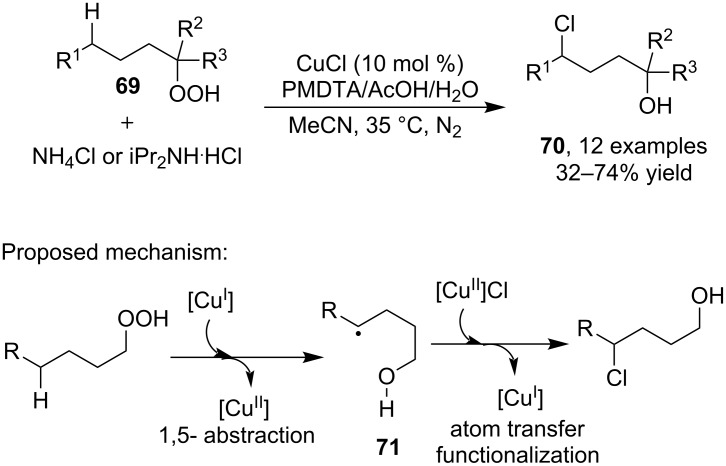
Copper-catalyzed remote C–H chlorination of alkyl hydroperoxides.

Compared with the C(sp^2^)–H bond, the C(sp^3^)–H bond is the less acidic one and is therefore known as the most challenging chemical bond for direct activation. Consequently, the examples on copper-catalyzed halogenation of inactive C(sp^3^)–H bond remained barely explored. In 2010, Ball and Kundu [68] developed a protocol of remote C–H chlorination of alkyl hydroperoxides by means of copper catalysis. As displayed in Scheme 23, the alkyl hydroperoxides **69** and proper chlorine source (NH_4_Cl or iPr_2_NH·HCl) could couple each other in the presence of CuI and *N*,*N*,*N*′,*N*′′,*N*′′-pentamethyldiethylenetriamine (PMDTA) to provide γ-chlorinated alcohols **70** via an intramolecular redox process. To enable the transformation, CuCl promoted the 1,5-H abstraction and atom transfer process in the form of SET via free radical **71**.

**Scheme 24:**
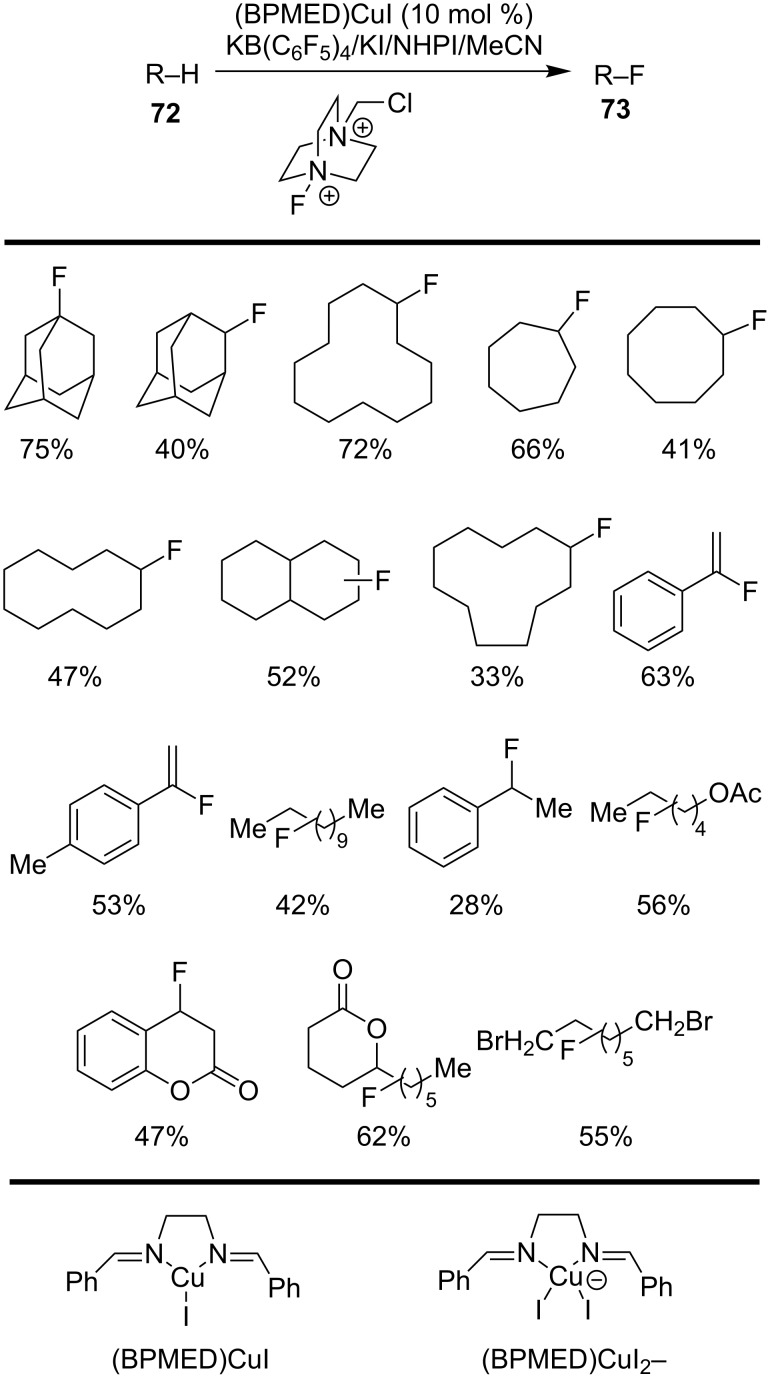
Copper-catalyzed C–H fluorination of alkanes.

In 2012, Lectka and co-workers [69] reported an interesting C–H fluorination method for alkynes **72** via a Cu-catalyzed aliphatic C(sp^3^)–H functionalization. Monofluorinated products **73** were obtained by employing a catalytic system consisting of (BPMED)CuI (copper(I) bisimine complex), *N*-hydroxyphthalimide (NHPI), KB(C_6_F_5_)_4_ and KI. The protocol allowed the selective fluorination of various substrates, including cycloalkanes and benzylic compounds using commercially available Selectfluor as fluorine source. According to the obtained results, the presence of KB(C_6_F_5_)_4_ as the phase-transfer catalyst could accelerate the reaction rate and enhance the yield of the products. On the other hand, the KI could promote the formation of the active cuprate species (BPMED)CuI_2_^−^, thus allowing less reactive substrates to convert smoothly (Scheme 24).

**Scheme 25:**
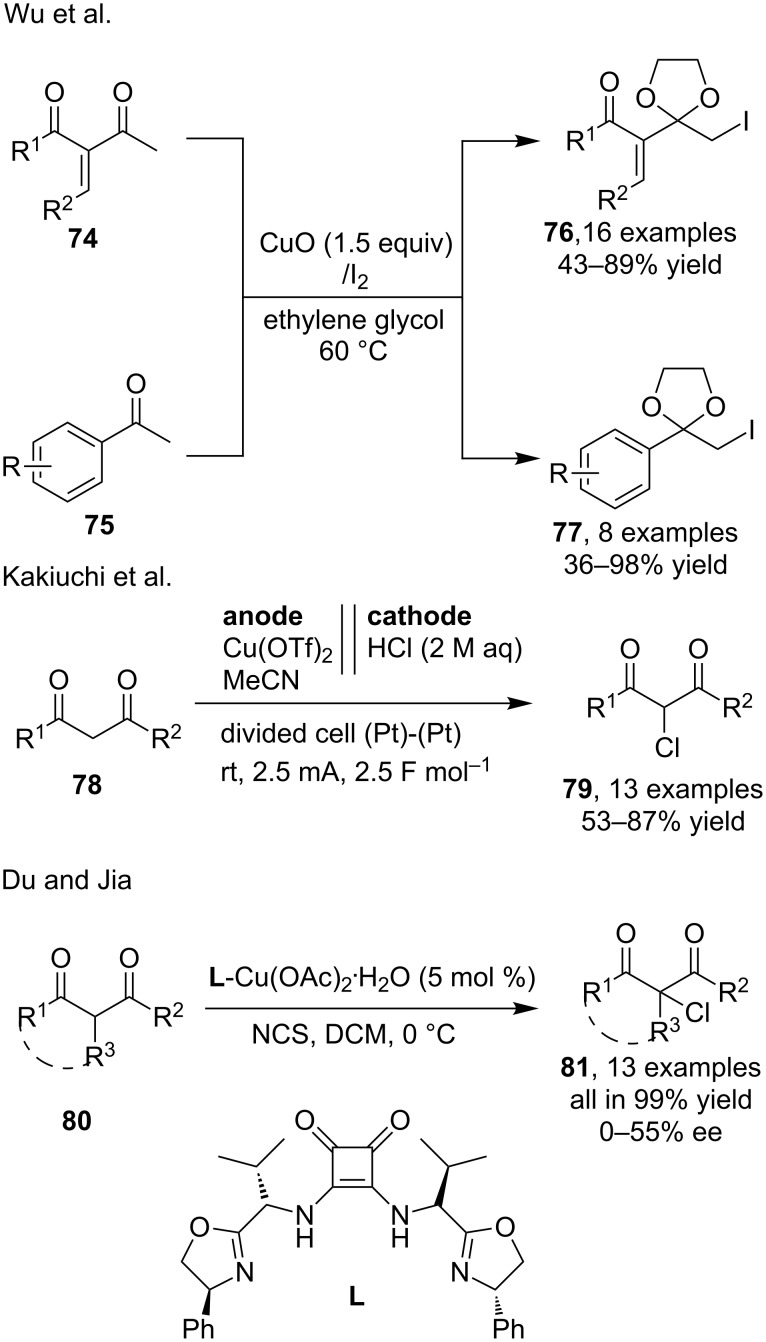
Copper-catalyzed or mediated C–H halogenations of active C(sp^3^)-bonds.

Besides catalyzing the halogenation of inactive alkane substrates via a typical C–H activation, copper catalysis also exhibited important application in the electrophilic halogenation of some active methylene substrates such as ketones or esters. Although these active substrates were known to be capable of undergoing α-C–H halogenations under simple acidic or basic conditions, the problem of unsatisfactory selectivity between mono- and multihalogenation or utilization of operationally unfriendly halogenating reagents were confronted frequently and thus prevented the practical application of these metal-free methods. The application of copper catalysts was found as effective solution to some of these problems. For example, Wu et al. [70] reported the efficient synthesis of α-iodoketals **76** and **77** via CuO-mediated selective mono-iodination of diketones **74** and methylketones **75** in the presence of molecular iodine, respectively. The tandem transformation of a carbonyl acetalization and a iodination in sustainable ethylene glycol under mild heating provided a practical approach in the synthesis of useful protected α-haloketones (Scheme 25). Recently, Kakiuchi and co-workers [71] successfully achieved the selective mono-α-chlorination of β-keto esters/amides and 1,3-diketone **78** by employing an electrochemical synthesis via a catalysis by means of Cu(OTf)_2_. The synthesis of chlorinated carbonyl products **79** were acquired in a divided cell using aqueous HCl as chlorine source (Scheme 25). On the other hand, Du and Jia [72] developed a route for the asymmetric chlorination of similar carbonyl substrates **80** via copper-catalyzed asymmetrical α-chlorination in the presence of a chiral ligand. The yield of all products **81** was excellent, and the enantioselectivity, however, was generally moderate (Scheme 25).

## Conclusion

Due to the widespread application of halogenated chemicals in organic chemistry, the synthesis of halogenated compounds via direct halogenations of C–H bonds is amongst the most important issue of modern organic synthesis. On the basis of the traditional electrophilic substitution reaction, the occurrence of powerful new synthetic strategies such as transition metal-catalyzed C–H activation brought new opportunities to the synthesis of more diversely halogenated products by enabling the halogenation of more challenging substrates by more selective transformations. Based on the summarization in this review, it can be found that magnificent advances were made in the copper-catalyzed halogenation, allowing much more options towards the synthesis of halogenated products. On the other hand, it also should be noted that most known literatures on the area focus on the conversion of arene C–H bonds. Although elegant works on alkene C–H bond and C(sp^3^)–H bond halogenation were also available in literature, the rare overall availability and not universal scope of application, however, demonstrated the remaining challenges in this research area. In addition, the unavailability of a practical copper-catalyzed halogenation of alkyne C(sp)–H bonds is also an issue requiring urgent attention.
